# Identifying irregular gasoline and ethanol blends in Brazil using a Love-wave sensor

**DOI:** 10.1098/rsos.252243

**Published:** 2026-10-21

**Authors:** Dailan de Jesus Pereira Bernardes, Ewaldo E. Carvalho Santana, Sergey Balashov, Raimundo Carlos, Allan Kardec, Paulo Fernandes Da

**Affiliations:** ^1^ Electrical Engineering, Sousândrade Foundation for Supporting the Development of the Federal University of Maranhão, São Luís, Brazil; ^2^ Electrical Engineering, Federal Institute of Education Science and Technology of Maranhão, São Luís, Brazil; ^3^ Engenharia da Computação, Universidade Estadual do Maranhão, São Luís, Brazil; ^4^ Nanoestruturas, CTI, Campinas, Brazil; ^5^ Engenharia Elétrica, Universidade Federal de Campina Grande, Campina Grande, Brazil; ^6^ Engenharia Elétrica, Universidade Federal do Maranhão, São Luís, Brazil

**Keywords:** Love-wave sensors, liquid analysis, irregular mixtures in fuels, gasoline and anhydrous ethanol fuel

## Abstract

The quality control of commercial fuels is of paramount importance for ensuring regulatory compliance, engine performance and consumer protection. In Brazil, the composition of gasoline and anhydrous ethanol fuel blends is subject to specific regulatory requirements, and deviations from the prescribed proportions may compromise fuel quality and facilitate economically motivated adulteration. This study proposes a preliminary methodology for the identification of irregularities in binary gasoline and anhydrous ethanol fuel blends based on a Love-wave sensor. The proposed approach exploits the multiphysics response of the sensor to variations in the physicochemical properties of the liquid mixtures, which are directly influenced by their volumetric composition. The results demonstrate that changes in the proportions of the constituent liquids produce distinguishable variations in the sensor response, enabling the identification of different blend compositions. The proposed methodology therefore demonstrates the feasibility of employing Love-wave sensors as a rapid and potentially field-deployable screening tool for the preliminary assessment of irregular fuel blends in Brazil. Such an approach may contribute to more efficient fuel quality surveillance by reducing analysis time and operational costs while supporting subsequent confirmatory analyses using conventional laboratory techniques.

## 1. Introduction

Fuel quality monitoring has traditionally relied on laboratory-based analytical techniques, including gas chromatography (GC), gas chromatography–mass spectrometry (GC–MS), near-infrared (NIR) spectroscopy, Raman spectroscopy, nuclear magnetic resonance (NMR), atomic absorption spectroscopy (AAS) and density or viscosity measurements [1–22]. These methods provide excellent analytical accuracy and are widely accepted as reference techniques for fuel certification. However, they generally require expensive instrumentation, specialized laboratory facilities, trained personnel and relatively long analysis times, making them unsuitable for rapid on-site inspections.

To overcome these limitations, several portable sensing technologies have been investigated for fuel quality assessment. Capacitive sensors, cataluminescence-based sensors and optical fibre sensors have demonstrated the capability to detect variations in gasoline and ethanol mixtures through changes in electrical or optical properties [23–25]. Although these approaches offer faster measurements and reduced operational costs, many still require specialized sensing materials, optical components or complex calibration procedures.

Surface acoustic wave (SAW) sensors constitute an attractive alternative because they combine high sensitivity, fast response, compact dimensions, low power consumption and compatibility with microfabrication technologies. These characteristics have led to their widespread application in chemical and biological sensing, environmental monitoring, industrial process control and liquid characterization.

From the viewpoint of chemical and liquid sensing, SAW sensors can be conveniently divided into two categories. The first employs a functional sensing layer deposited on the piezoelectric substrate to provide chemical selectivity through specific interactions with the target analyte. The second comprises coating-free sensors, in which the piezoelectric surface remains unmodified, and the sensor directly measures changes in the physical properties of the surrounding medium. Although this is not the conventional classification adopted in the SAW literature, it provides a convenient framework for comparing sensing strategies. Functionalized SAW sensors rely on selective coatings such as polymers, metal oxides, carbon nanomaterials, molecularly imprinted polymers or biological recognition elements. These materials enable highly selective detection of target analytes, often at parts-per-billion concentrations, making them suitable for gas sensing, environmental monitoring and biosensing applications [26–29].

Coating-free SAW sensors, by contrast, detect variations in bulk properties including electrical conductivity, dielectric permittivity, density and viscosity. Their response results from direct interaction between the acoustic wave and the adjacent liquid through acoustoelectric coupling, viscous loading and mass loading, producing measurable changes in insertion loss, phase velocity, propagation delay or resonance frequency. Because coating-free sensors rely on physical rather than chemical interactions, they cannot identify individual chemical species. However, this limitation is unimportant in many industrial quality-control applications, where the objective is to verify compliance with specified physical-property limits rather than determine the composition of contaminants [26,30].

Fuel quality assessment is a representative example. Commercial fuels are regulated according to acceptable ranges of conductivity, dielectric permittivity, density, viscosity and other physicochemical properties. Adulteration or degradation modifies one or more of these parameters, even when the adulterant consists of an unknown or complex chemical mixture. Consequently, rapid measurement of bulk physical properties is often more useful for quality control than complete chemical identification, and product acceptance is based on compliance with standardized specifications [31–34].

For such applications, coating-free SAW sensors offer important advantages. The absence of a functional coating eliminates problems associated with coating ageing, poisoning, delamination and chemical instability while improving resistance to aggressive liquids such as gasoline, diesel fuel, ethanol and biodiesel. The bare piezoelectric surface can be readily cleaned after each measurement, enabling repeated long-term use with minimal degradation of sensor performance. These characteristics make coating-free SAW sensors particularly attractive for robust industrial monitoring and rapid fuel quality assessment [26,30].

Among the various SAW configurations, Love-wave sensors are particularly suitable for liquid sensing applications. A Love-wave device consists of a piezoelectric substrate coated with an acoustic waveguiding layer that confines the acoustic energy near the surface, significantly enhancing sensitivity to surface perturbations. The guiding layer also electrically isolates the interdigital transducers (IDTs) from the liquid, allowing stable operation in conductive media. Consequently, Love-wave sensors exhibit excellent sensitivity to changes in density, viscosity, electrical conductivity, permittivity and mass loading, making them highly suitable for liquid analysis.

In this work, a Love-wave SAW sensor of the second group is proposed for the rapid detection of quality irregularities in Brazilian commercial gasoline. Unlike conventional laboratory techniques, the proposed device enables real-time measurements using small sample volumes while maintaining a compact, portable and low-cost architecture. The sensor operates without a chemically selective sensing film, exploiting only the intrinsic physicochemical differences between gasoline and ethanol mixtures. This approach provides a promising platform for rapid field inspection and fuel quality monitoring.

## 2. Material and methods

In this section, the analysed commercial fuel products are described along with their mechanical (density and viscosity) and electrical (permittivity and conductivity) properties. The sensor employed in this application is also presented, as well as the testing methodology, which was conducted in the laboratory but can also be performed under field conditions.

### 2.1. Sample preparation

The physicochemical properties of the commercial fuels investigated in this study are summarized in table 1. Density, dynamic viscosity and relative dielectric permittivity were measured at 20°C. Density was determined using a DMA 4500 density metre (Anton Paar GmbH), dynamic viscosity using a Brookfield DV-III Ultra rheometer and relative dielectric permittivity using a Keysight E4991B impedance analyser. Although electrical conductivity is also an important fuel property, it was not measured because suitable instrumentation was unavailable for all investigated liquids.

**Table 1.** Physicochemical properties of the commercial fuels investigated (20°C).

fuel	density (kg m^−3^)	absolute viscosity (cP)	relative dielectric permittivity
commercial fuels			
gasoline	750	0.89	5
anhydrous ethanol fuel	789	1.14	25

To prepare adulterated samples, gasoline was blended with anhydrous ethanol at concentrations of 5%, 30% and 40% (v/v). These concentrations were selected to produce systematic variations in the bulk properties of the fuel while maintaining well-defined and reproducible compositions. The physicochemical properties of the prepared mixtures are summarized in table 2. All measurements were performed under the same experimental conditions used for the commercial fuels.

**Table 2.** Physicochemical properties of the gasoline–ethanol mixtures prepared in this study (20°C).

fuel mixture	density (kg m^−3^)	absolute viscosity (cP)	relative dielectric permittivity
commercial fuels			
gasoline with 5% anhydrous ethanol by volume (v/v)	752	0.9	6
gasoline blended with 30% anhydrous ethanol by volume (v/v)	761	0.95	7
gasoline blended with 40% anhydrous ethanol by volume (v/v)	765	1.01	10

The aim of this study is to demonstrate the feasibility of using Love-wave sensors for the detection of irregular mixtures in commercial fuels. In all cases, both the mechanical properties (density and viscosity) and electrical properties (relative permittivity and conductivity) of the mixtures were altered, disturbing the acoustic wave propagation and electromechanical response of the sensor. These combined effects enrich the sensor signal, allowing for the reliable discrimination and identification of different mixtures based on their multiphysics signatures, in other words, each mixture exhibits a unique combination of electrical and mechanical properties, resulting in distinct perturbations of the sensor response. The values listed in table 2 were measured under the same conditions as those in table 1.

### 2.2. Sensor used in this application

In general, Love-wave devices consist of a piezoelectric substrate coated with a thin layer known as the guiding layer. This layer slows down the acoustic waves and confines them to the surface, thereby increasing the sensitivity of the sensor. The guided layer is deposited on top of the crystal surface with IDTs, effectively isolating them from the liquid medium, which prevents them from being shorted by the liquid. Because the guiding layer is made of an insulating material, the IDTs remain electrically isolated from the liquid medium, which favours their use in such environments. Figure 1 illustrates a device with the basic configuration of a Love-wave sensor [31–37].

**Figure 1.**
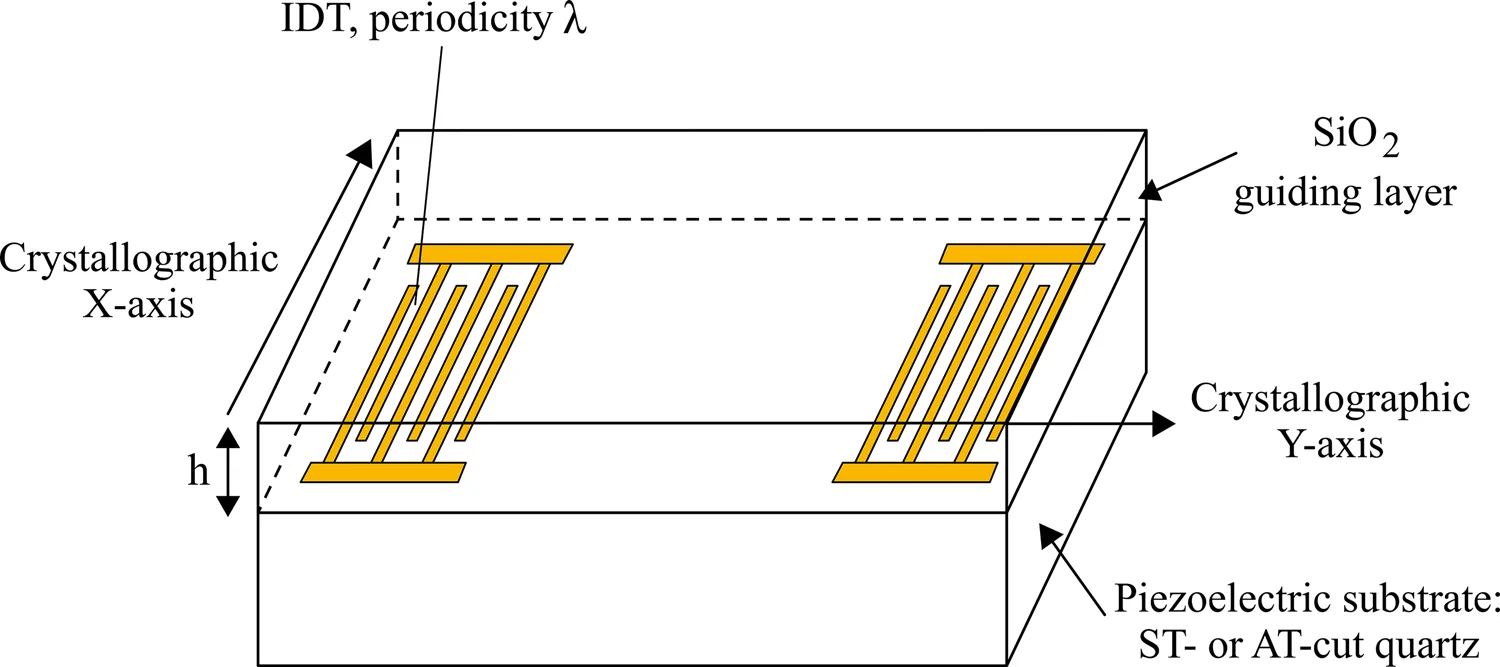
Structure of a Love-wave sensor.

A Love-wave SAW delay-line sensor was employed in this application. The device consists of a 500 µm-thick ST-90°X quartz substrate on which IDTs were fabricated. The IDTs were made of aluminium and designed with an acoustic wavelength of 40 µm (corresponding to the distance between adjacent IDT fingers connected to different electrical potentials), resulting in a centre frequency of 117 MHz. A 4-µm-thick silicon dioxide (SiO₂) waveguiding layer was deposited over this structure, serving both as a guiding layer for the Love-wave propagation and as an insulating layer for the IDTs, preventing the liquid under test causing a short circuit in them. The ratio between the waveguiding layer thickness and the acoustic wavelength is 0.1. This configuration promotes the confinement of acoustic energy near the sensor surface, thereby enhancing its sensitivity to surface perturbations. The sensor structure is illustrated in figure 2.

**Figure 2.**
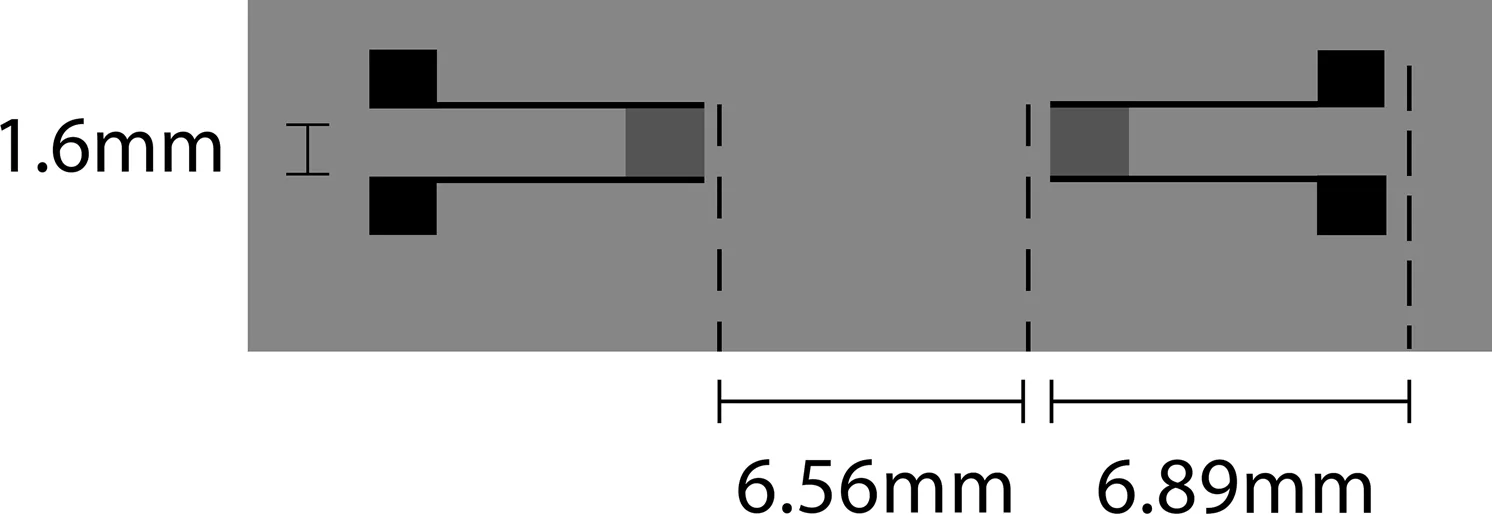
117 MHz Love-wave sensor.

In this configuration, the acoustic waves propagate at an approximate Love-wave velocity of *v*_Lw_ =  4680 m s^–1^. The time required, *t*_d_, for the acoustic wave to propagate over the distance L between the IDTs can be determined using equation (2.1):

(2.1) $$t_{d}=\frac{L}{v_{Lw}},$$

For the device used in this study, the distance was *L* = 6.56 mm, resulting in an intrinsic acoustic propagation time of approximately 1.4 µs. The overall response time of a measurement system such as the one proposed in this work also depends on additional factors, including the intrinsic noise of the instrumentation and the stabilization of the liquid mixture on the sensing surface. Although this response time was not experimentally measured, it is expected to be no longer than a few seconds.

The influence of the mechanical properties (viscosity and density) of the liquid, as reported in [35,36], on the phase velocity and central frequency of the propagating Love waves, was analysed. Additionally, variations in the electrical properties of the liquid (relative permittivity and conductivity), as indicated in [36,38], were considered because of their impact on the sensor electrical response. Figure 3 depicts the test cell used to localize the liquid over the sensitive propagation path between the IDTs and directly on top of the IDTs. Depositing liquid between the IDTs primarily perturbs the acoustic wave propagation, affecting the amplitude and phase, whereas placing liquid over the IDTs modulates the electrical conditions of the device, enriching the overall multiparametric sensor response. This approach enables the discrimination and identification of different liquid mixtures based on combined mechanical and electrical effects.

**Figure 3.**
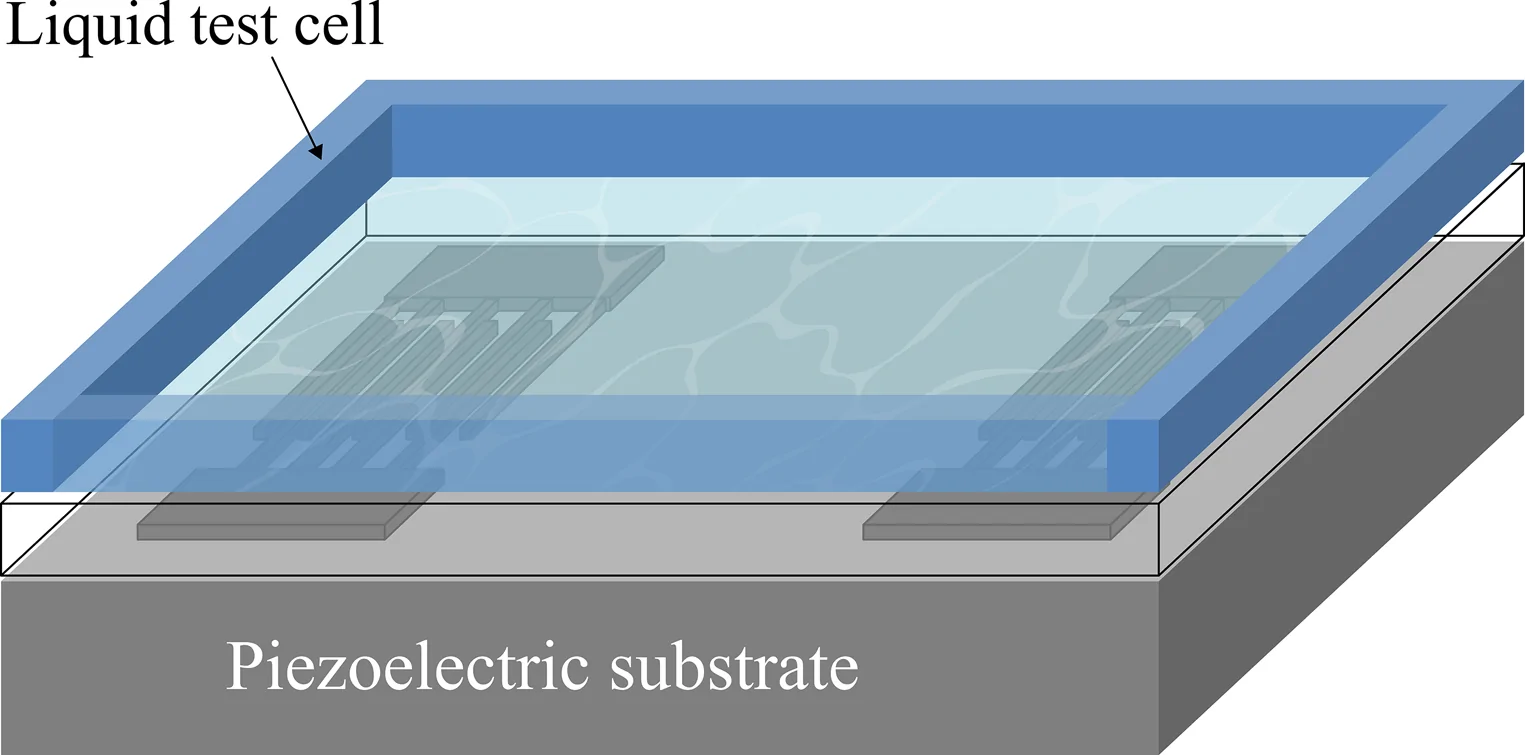
Sensor with liquid under analysis.

### 2.3. Open loop measurement method

An open-loop measurement (OLM) configuration based on a vector network analyzer (VNA) was employed to characterize the Love-wave sensor. The VNA measures the scattering parameters (S-parameters), which describe the amplitude and phase relationships between the incident and reflect electromagnetic waves at the device under test. Unlike closed-loop architectures, where SAW-induced phase variations are converted into frequency shifts by incorporating the sensor into an oscillator feedback loop, the OLM directly measures the amplitude and phase responses of the device. Although oscillator-based systems generally provide higher sensitivity, their performance may be degraded by frequency instabilities under field conditions, often requiring additional compensation circuitry or digital correction algorithms [34]. By contrast, modern compact VNAs, such as the NanoVNA, provide adequate frequency stability at low cost, making the OLM an attractive alternative for portable sensing systems.

The sensitivity OLM parameters depend on the location where the liquid perturbs the acoustic wave. As illustrated in figure 3, the acoustic propagation path between the two IDTs is considerably longer than the transducer length. Consequently, changes occurring along the delay line accumulate over the entire propagation distance, producing significant variations in insertion loss and phase. By contrast, the centre frequency is primarily determined by the SAW velocity within the IDTs, where the acoustic wavelength is defined by the electrode periodicity. Since the SAW velocity shift owing to additives is small, the resulting centre-frequency shift is expected to be much smaller than the corresponding changes in insertion loss and phase.

Accordingly, two scattering parameters were analysed. The reflection coefficient, *S*₁₁, represents the power reflected at the input port and provides information about the electrical impedance of the sensor. The forward transmission coefficient, *S*₂₁, represents the power transmitted between the input and output transducers and enables the evaluation of insertion loss, phase shift and resonance characteristics. Figure 4 presents the open-loop measurement set-up used for sensor characterization.

**Figure 4.**
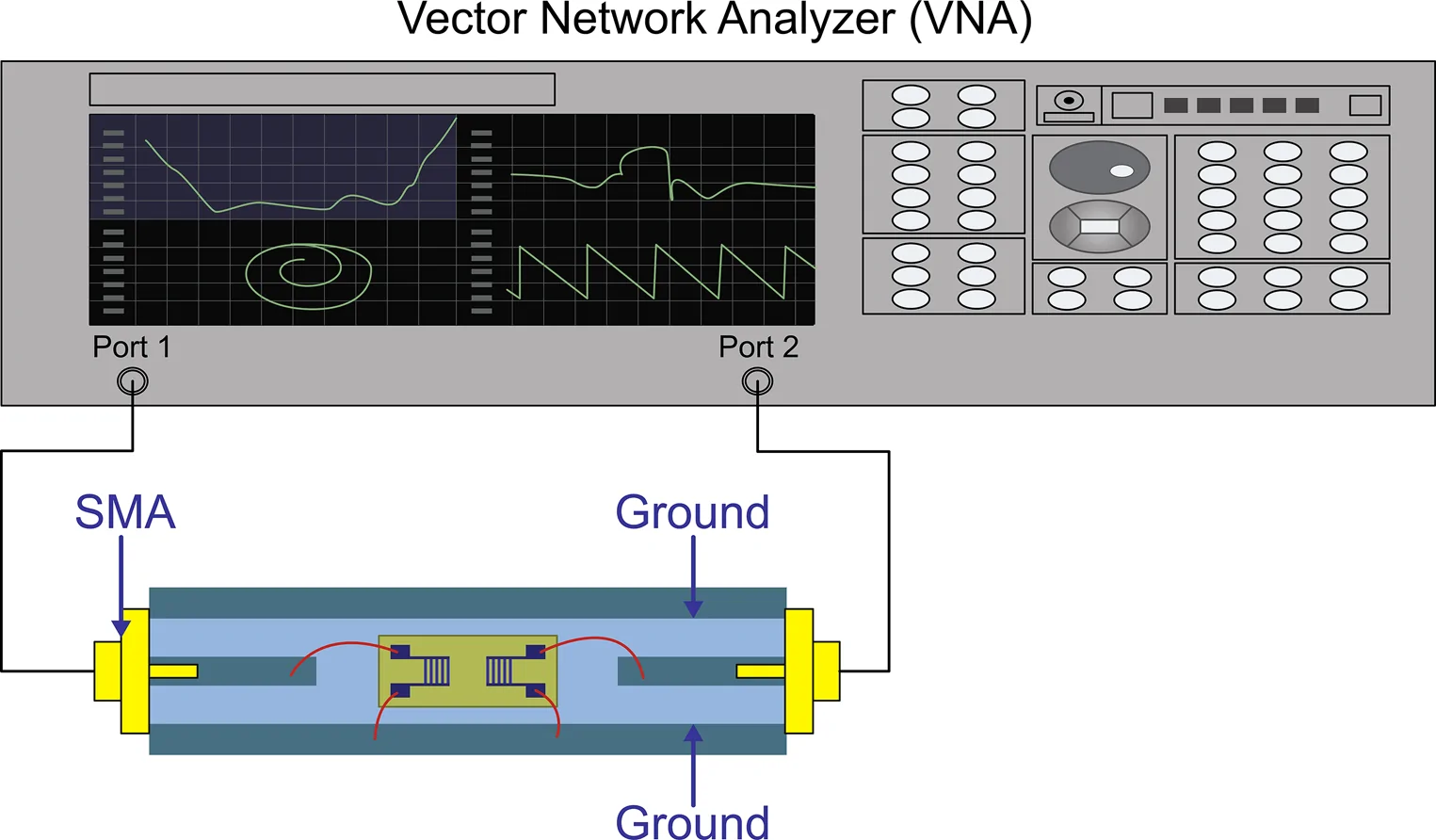
SAW sensor S-parameter measurement with VNA.

A polydimethylsiloxane-based liquid test cell was employed to confine the liquid sample on the sensing area, including the region above the SiO₂-coated IDTs, which protected the electrical contacts from fluid exposure and the device edges to prevent acoustic wave reflections. A 200 µl volume of each liquid sample was deposited by the drop-casting method using a micropipette. After each liquid test, the Love-wave sensor was cleaned sequentially with isopropyl alcohol, acetone and deionized water, followed by a hot air drying step. Measurements were carried out for all the liquid samples listed in tables 1 and 2. The objective was to discriminate the commercial liquids based on the effects of their mechanical (viscosity η and density ρ) and electrical (permittivity ε₀ and conductivity σ₀) properties on the device response. All measurements were performed at 20°C using two different VNAs: a LibreVNA (Seesii) and an ENA E5061B (Keysight). No significant variations were observed between the results obtained with both VNAs. Figure 5 illustrates the measurement set-up used in this work.

**Figure 5.**
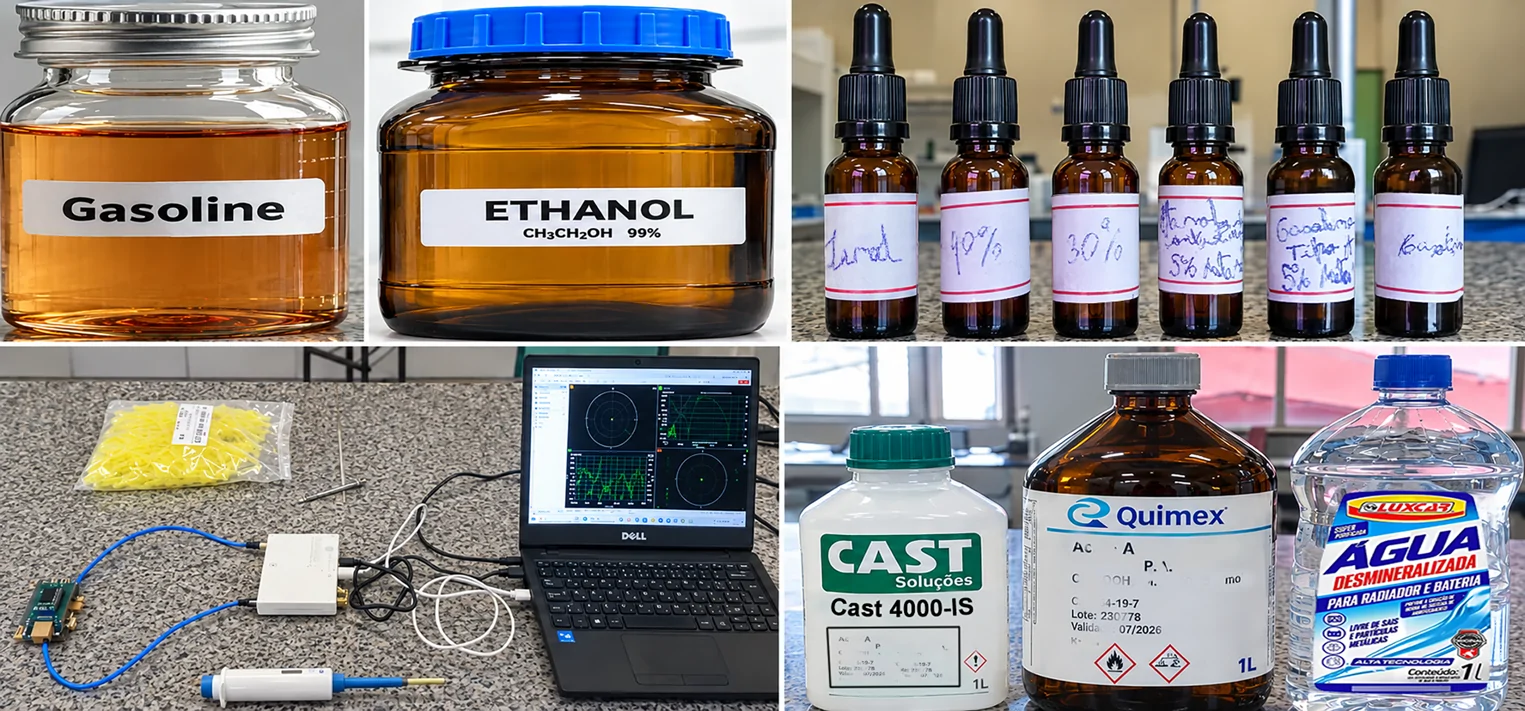
Measurement set-up and cleaning liquids.

## 3. Results

In this section, the S-parameter responses of the Love-wave device are presented. In particular, the magnitude and phase of *S*₂₁, as well as the magnitude of *S*₁₁, were analysed. These responses allow distinguishing among the tested liquid samples, because each liquid induces distinct perturbations on the acoustic wave propagation and, consequently, on the electrical characteristics of the device. In [39], equations are presented demonstrating the influence of liquid viscosity and density on the sensitive region of the Love-wave device, evidencing the perturbation of the insertion loss of the sensor. In [36], based on the analysis of the input power, the expression for the insertion loss parameter *S*_21_ is derived, as shown in equation (3.1):

(3.1) $$S_{21}=\;Asinc(X^{2})e^{-j\omega t_{d\;}},$$

where *A* denotes the mechanical damping caused by the medium to the electroacoustic wave along its propagation path between the IDTs, *t*_**d**_  represents the time required for the Love wave to travel along the acoustic path, also referred to as the time delay, which is directly related to the phase shift of the propagating wave on the sensor surface and is influenced by the viscosity and mass loading of the analytes deposited on the device surface. The term *X* corresponds to the normalized frequency parameter, defined by

(3.2) $$X\;=\;\pi N\frac{f-f_{r}}{f_{r}},$$

where *N* is the number of periods of the IDT and *f_r_*- is the central frequency determined by the period of the IDT structure and Love-wave velocity. Thus, based on equation (3.2) and according to the authors’ analysis, it was observed that higher viscosity and density of the liquid deposited on the sensor surface lead to increased insertion losses; that is, the *S*_21_ modulus parameter exhibits smaller values.

By analysing equation (3.2) for the Love-wave device investigated in this study, it can be observed that the exponential term represents the phase delay, while its magnitude is always equal to unity. Consequently, this term does not affect the magnitude of the insertion loss. Moreover, since the parameter (X) is very small and close to zero around the operating frequency, the cardinal sine function approaches unity. Therefore, the insertion loss is predominantly determined by the mechanical damping coefficient, which accounts for the attenuation experienced by the acoustic wave during its propagation between the IDTs.

The mechanical damping originates from the interaction between the Love wave confined within the guiding layer and the liquid in contact with the sensing surface. From a physical standpoint, the acoustic energy carried by the surface wave is partially dissipated into the liquid because of its mechanical properties, primarily viscosity and density. Consequently, liquids with higher viscosity and density induce greater acoustic energy dissipation, leading to increased insertion losses. Accordingly, the mechanical damping coefficient, and therefore the insertion loss, is strongly correlated with the term $\sqrt{\eta \rho }$, where η denotes the viscosity and ρ is the liquid density. This relationship has been widely reported for Love-wave sensors operating in liquid media and constitutes the dominant mechanical loading mechanism governing acoustic attenuation.

The sensor response is analysed using a VNA, which measures the scattering parameters based on the incident and reflects electromagnetic waves. Equation (3.3) is employed to convert the scattering parameter *S*_11_ into the input impedance parameter *Z*_11_, a representation that is more commonly used for the electrical characterization and interpretation of electronic and RF devices. This relationship is explicitly presented to facilitate the understanding of the proposed methodology and to complement the conventional scattering-parameter analysis by providing additional insight into the electromechanical interaction between the acoustic wave and the liquid under test. Moreover, the impedance-based analysis demonstrates the sensitivity of the sensor to the electrical properties of the analysed liquid, particularly its dielectric characteristics, as previously reported in [38], thereby providing a more comprehensive interpretation of the sensor response:

(3.3) $$\;Z_{11}=\;50\frac{1+S_{11}}{1-S_{11}}\;,$$

The parameter *Z*_11_ is directly related to the input impedance of the SAW sensor, which can be mathematically represented as a complex quantity consisting of an equivalent series resistance and a capacitive reactance. The equivalent resistance is primarily associated with the conversion of electrical energy into acoustic waves and with the intrinsic electrical losses of the device, whereas the reactance is related to the capacitive effects of the IDTs and the energy stored within the transducer structure. The latter component is of particular interest in this work because it is expected to contribute to the discrimination of different liquid mixtures through variations in their dielectric permittivity, which directly affect the capacitive reactance of the sensor. Consequently, the impedance response provides complementary information to the conventional scattering-parameter analysis, enabling a more comprehensive electrical characterization of the sensing mechanism.

When a liquid is deposited onto the sensor surface, its mechanical and electrical properties modify the acoustic wave propagation conditions, thereby changing the electromechanical coupling efficiency and the amount of energy reflected at the sensor input. These changes are directly reflected in the measured *S*_11_ parameter and, consequently, in the input impedance *Z*_11_. Therefore, the proposed impedance-based analysis aims to demonstrate the importance of these interactions, particularly for Love-wave sensors, whose enhanced sensitivity arises from the strong confinement of acoustic energy within the guiding layer. This characteristic makes Love-wave devices especially suitable for detecting subtle variations in both the electrical and mechanical properties of liquid media, thereby improving the discrimination capability between different liquid mixtures.

### 3.1. Gasoline blended with anhydrous ethanol

In this section, the variation in the sensor response owing to the deposition of gasoline, anhydrous ethanol fuel and gasoline and ethanol mixtures in volumetric ratios of 30% and 40% is analysed. In [37], the authors conducted an experimental study that demonstrated the variation in the insertion loss as a function of the parameter $\sqrt{\eta \rho }$. Table 3 presents the calculated values of this parameter for the liquids analysed.

**Table 3.** Liquid parameters under test.

liquid sample	$\sqrt{\eta \rho }$
gasoline	25.84
gasoline blended with 30% anhydrous ethanol by volume	26.89
gasoline blended with 40% anhydrous ethanol by volume	27.79
anhydrous ethanol fuel	29.99

Figure 6 presents the insertion loss curves obtained for the different liquids analysed in this section. Figure 6a shows the insertion loss magnitude, whereas figure 6b illustrates the corresponding phase variation resulting from the perturbation induced by the liquids on the sensor sensitive surface. The insertion loss variation is characterized by the *S*_21_ parameter, which is directly associated with the energy transfer of the guided surface acoustic wave as it interacts with the liquid in contact with the sensing surface. The liquid deposited on the guiding layer increases the dissipation of acoustic energy owing to mass loading and viscous effects, in addition to the influence of its electrical properties. Consequently, the amplitude of the propagating acoustic wave is attenuated, leading to an increase in the insertion loss.

**Figure 6.**
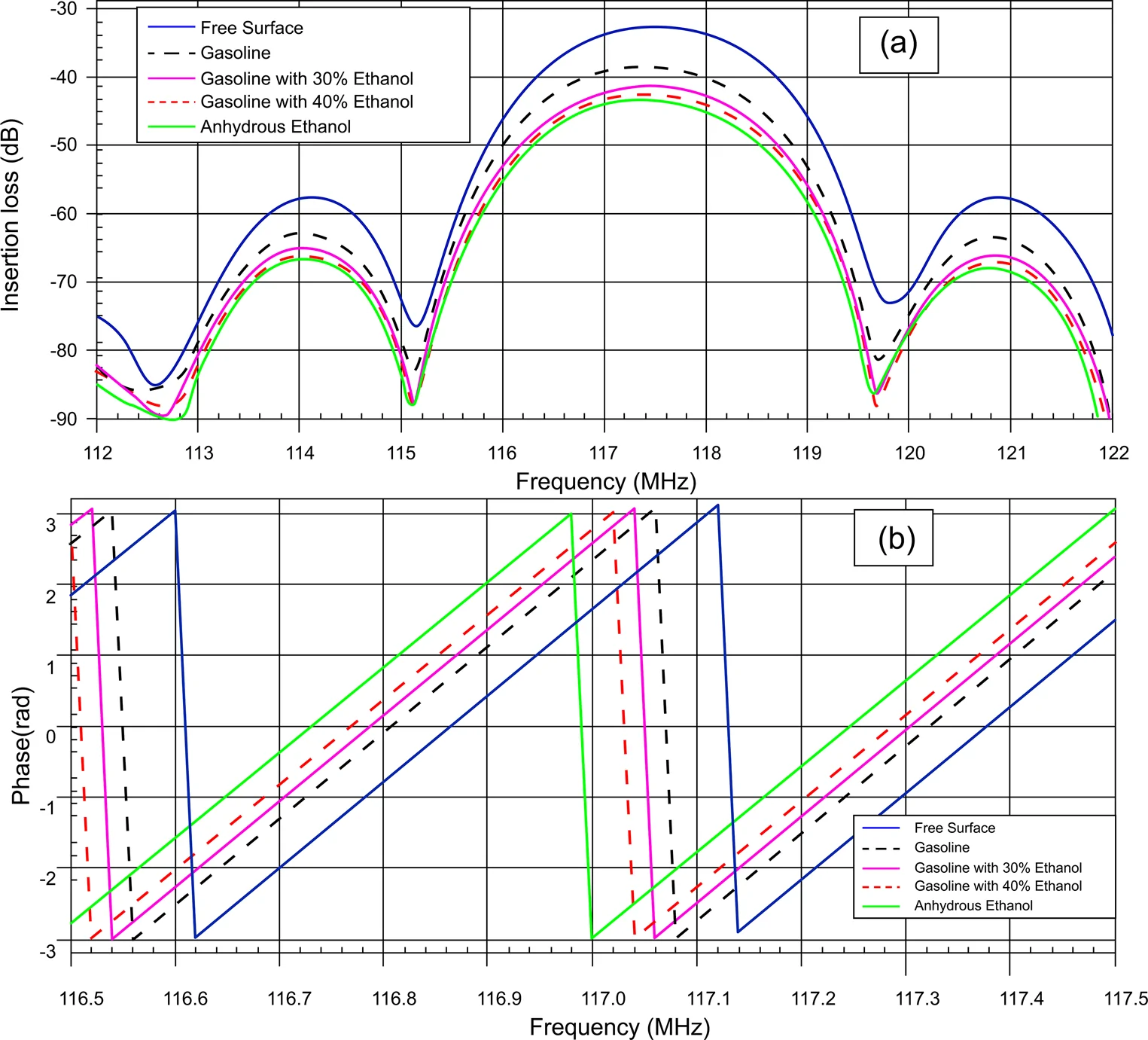
Love-wave sensor transmission response *S*_21_ with gasoline, ethanol and mixtures, insertion loss (a) and phase (b).

Figure 6a does not exhibit noticeable resonance frequency shifts because, as demonstrated in [27,38], such shifts are primarily governed by variations in the $\sqrt{\eta \rho }$. For the liquids investigated in this study, the variations in this parameter are insufficient to produce measurable frequency deviations within the frequency scale presented. However, the phase response shown in figure 6b reveals discernible phase shifts, which are intrinsically associated with resonance frequency variations. This observation indicates that frequency shifts are indeed present, although they are in order of only a few kilohertz and, therefore, are not visually distinguishable in the frequency response of figure 6a.

In the present study, resonance frequency variation was not considered the primary sensing metric because the objective is to exploit the enriched sensor response rather than relying solely on frequency tracking. Specifically, this work aims to demonstrate that the dielectric properties of the analysed liquid mixtures also induce measurable perturbations in the sensor response. These electrically induced effects, together with the conventional mechanical interactions, provide additional discriminatory information, thereby enhancing the capability of the Love-wave sensor to distinguish among the investigated liquid mixtures.

Figure 7 shows the *S*_11_ curves obtained for the different liquids analysed in this section, indicating that the experimental response agrees with the expected theoretical behaviour. As the permittivity values of the liquids increase as presented in table 2 the return loss also increases, confirming the direct influence of the permittivity on the *S*_11_ parameter. This confirms the feasibility of identifying these mixtures using this sensing device.

**Figure 7.**
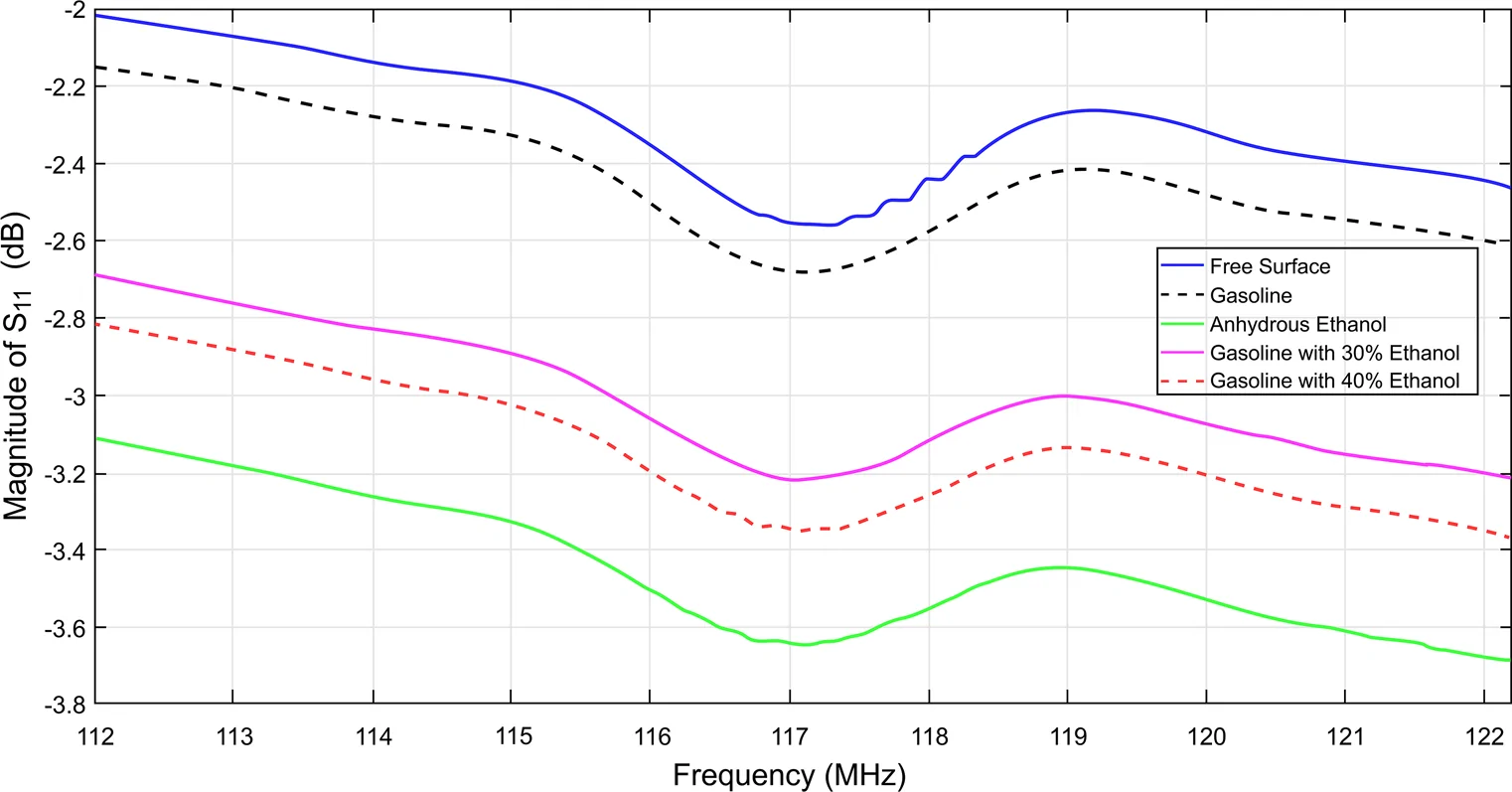
Love-wave sensor reflection response with gasoline, ethanol and mixtures, *S*_11_ module.

#### 3.1.1. Gasoline mixed with 5% anhydrous ethanol

In this section, the capability of the Love-wave sensor to identify a binary mixture of Type A gasoline and anhydrous ethanol containing 5% ethanol by volume was evaluated. The experimental results demonstrate that the proposed device can detect changes in the composition of the liquid mixture at this concentration level. Table 4 summarizes the values of the $\sqrt{\eta \rho }$ parameter calculated for the analysed samples.

**Table 4.** Liquid parameters under test.

liquid sample	$\sqrt{\eta \rho }$
gasoline	25.84
gasoline with 5% anhydrous ethanol by volume	26.02
anhydrous ethanol	29.99

Figure 8 presents the insertion loss responses obtained with the Love-wave sensor for pure gasoline, gasoline containing 5% (v/v) anhydrous ethanol and pure anhydrous ethanol deposited on the sensor's sensitive surface. Figure 8a shows the insertion loss magnitude, whereas figure 8b illustrates the corresponding phase response resulting from the interaction between the liquids and the sensing region.

**Figure 8.**
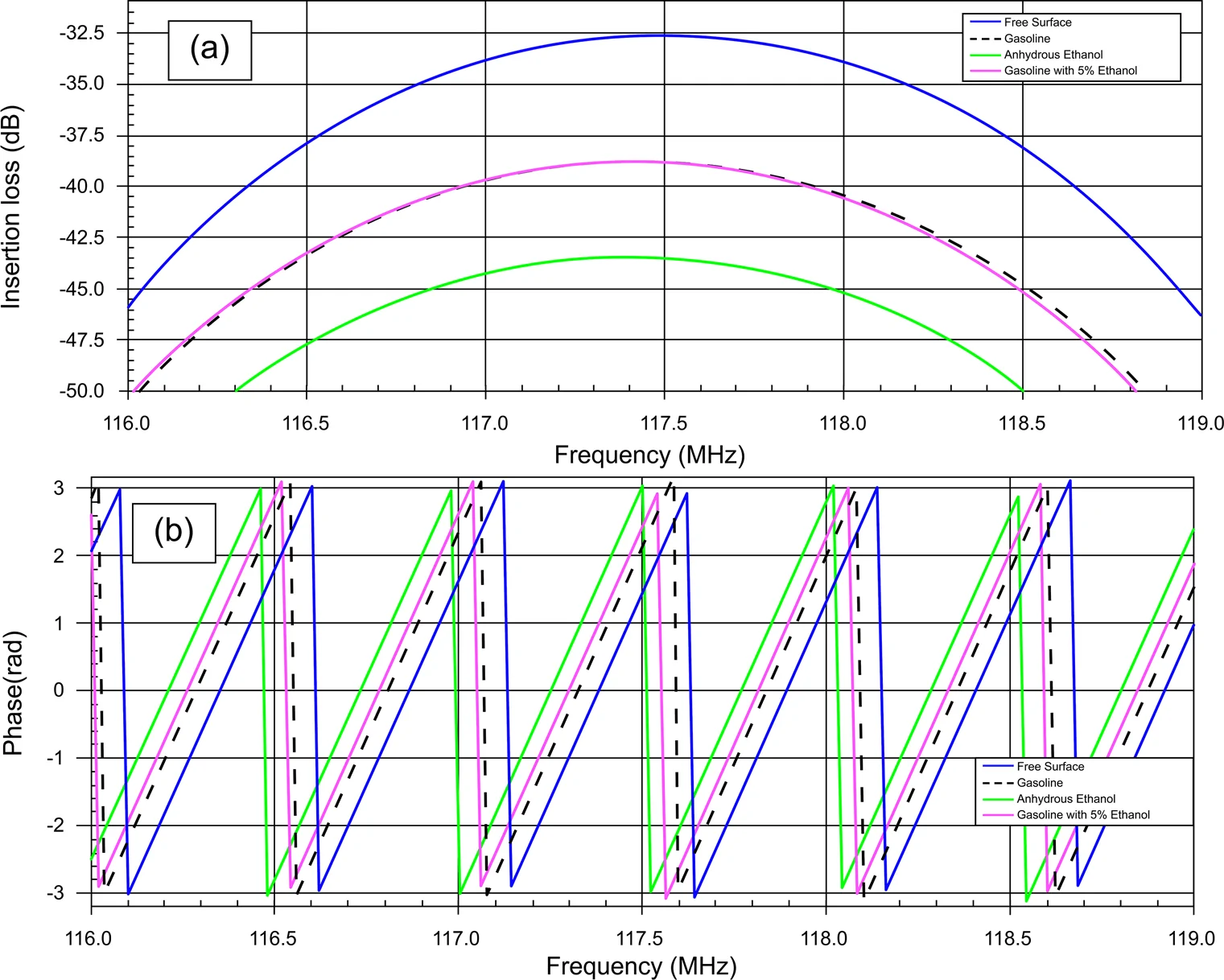
Love-wave sensor transmission response *S*_21_ with gasoline, methanol and mixture, insertion loss (a) and phase (b).

The purpose of this experiment was to evaluate the sensor's capability to detect small variations in the ethanol concentration, even though Brazilian commercial gasoline is legally required to contain 30% anhydrous ethanol by volume. Specifically, the feasibility of the proposed sensing approach was assessed for identifying a 5% deviation in ethanol concentration. The experimental results demonstrate that the proposed method is sufficiently sensitive for this application, since even such a small concentration difference produces measurable changes in the sensor response. As shown in figure 8a, the insertion loss exhibits only a slight increase when comparing pure gasoline with the gasoline and ethanol mixture containing 5% ethanol. Nevertheless, figure 8b reveals a clearly distinguishable phase variation, indicating that the phase response provides enhanced sensitivity to subtle changes in the composition of the liquid mixture.

Physically, an increase in the liquid viscosity hinders the oscillatory motion induced by the propagating acoustic wave. As a result, greater mechanical energy is dissipated through viscous damping, reducing the amplitude of the acoustic wave reaching the output IDT. The liquid density also plays a fundamental role in this process, as a higher density corresponds to a greater mass per unit volume, thereby requiring more energy for wave propagation along the acoustic path. In practice, these properties act simultaneously and are represented by the parameter $\sqrt{\eta \rho }$**,** which models the combined influence of density and viscosity on the insertion loss of the sensor. As expected, the experimental results exhibited a clear correlation with the increase in both density and viscosity, as summarized in table 5. These findings confirm that higher values of these parameters lead to increased insertion loss and larger phase variations, thereby demonstrating the capability of the Love-wave sensor to discriminate among the different liquid mixtures analysed.

**Table 5.** Variation of insertion losses with density and viscosity.

	gasoline	gasoline with 5% anhydrous ethanol	gasoline with 30% anhydrous ethanol	gasoline with 40% anhydrous ethanol	anhydrous ethanol
insertion loss in *f_r_* (dB)	−38.67	−38.81	−41.39	−42.37	−43.40
$\sqrt{\eta \rho }$	25.84	26.02	26.89	27.79	29.99

The relationship between the insertion loss and the term$\sqrt{\eta \rho }$ is corroborated by the results presented in table 5, where the insertion loss is observed to increase with increasing liquid viscosity and density. This behaviour demonstrates the sensitivity of the Love-wave sensor to these mechanical properties, confirming that variations in the visco-inertial loading imposed by different liquids directly affect acoustic energy dissipation during wave propagation. Consequently, the measured insertion loss provides a reliable parameter for discriminating among different liquid mixtures, thereby enabling their identification based on their distinct mechanical signatures.

Figure 9 presents the *S*₁₁ responses obtained for the different liquids investigated, demonstrating that the experimental results are consistent with the expected sensor behaviour. As the dielectric permittivity of the liquids increased, as summarized in table 2, a corresponding increase in the reflection losses was observed, confirming the direct influence of the liquid permittivity on the *S*₁₁ parameter. These findings demonstrate that the *S*₁₁ response provides complementary information for liquid characterization and can be effectively combined with the other measured sensor parameters to improve the discrimination and identification of the investigated mixtures.

**Figure 9.**
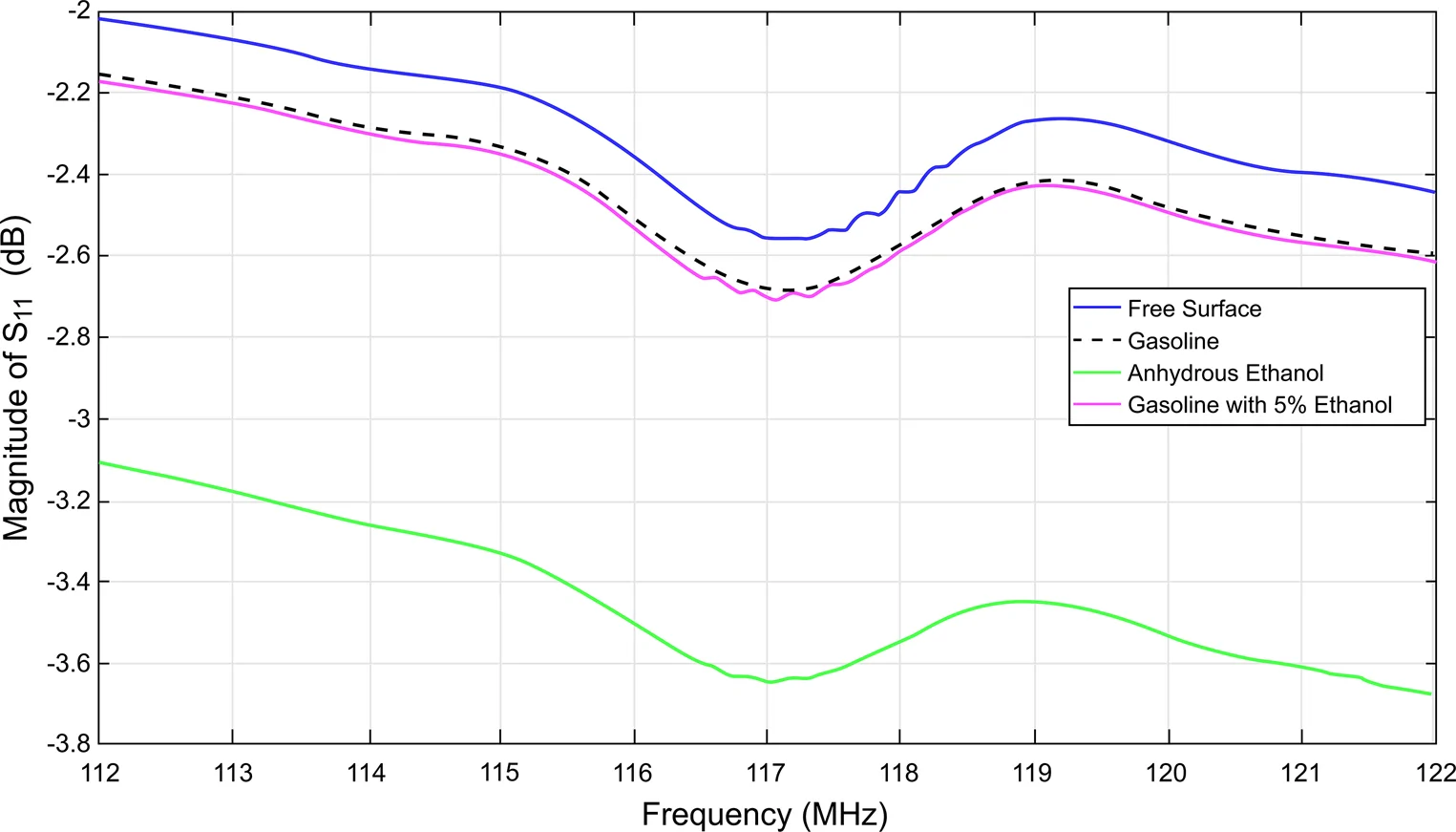
Love-wave sensor reflection response with gasoline, ethanol and mixture, module de *S*_11_.

Figures 8 and 9 collectively constitute the multiphysics signature of the analysed liquids, which is proposed as the basis for identifying each liquid mixture. Distinct mixtures produced different insertion loss, phase shift and reflection loss responses, indicating that each composition generates a unique combination of electromechanical perturbations in the Love wave sensor. The combined analysis of these response parameters is therefore expected to enable reliable discrimination and identification of the investigated mixtures, as each mixture exhibits a characteristic and unique multiphysics signature.

## 4. Discussion

In this section, the results obtained for the insertion loss (*S*₂₁) in both magnitude and phase, as well as for the reflection loss (*S*₁₁), are analysed for the sensor exposed to the different liquids under study. From the data presented in table 6, it can be observed that the most significant variations occur in the magnitude of the insertion loss, whereas the central frequency shift remains small. Nevertheless, the results obtained demonstrate the potential use of this device as a sensor for identifying the different mixtures analysed in this work, owing to the relevant variations in the insertion loss and perturbations in the sensor impedance characteristics. These perturbations, observed through the magnitude of the return loss (*S*₁₁), enabled the identification of changes in the volumetric proportion of the mixtures. The phase variations are not readily observable, as they are masked by the multiple phase rotations accumulated by the acoustic wave during its propagation along the acoustic path.

**Table 6.** Frequency response parameters of the sensor with perturbed surface.

	sensor with free surface	gasoline	gasoline with 5% anhydrous ethanol	gasoline with 30% anhydrous ethanol	gasoline with 40% anhydrous ethanol	anhydrous ethanol
frequency with lowest losses-*f_r_* (MHz)	117.5	117.5	117.36	117.36	117.36	117.36
insertion loss in *f_r_* (dB)	−32.77	−38.67	−38.81	−41.39	−42.37	−43.40
phase(rad) in *f_r_*	3.14	3.14	3.14	3.14	3.14	3.14
magnitude of *S*₁₁ in *f_r_*	−2.53	−2.66	−2.69	−3.21	−3.34	−3.63

Using the sensor with free surface as a reference, equation (4.1) expresses the relative variation of the insertion loss, whereas equation (4.2) represents the relative variation of the reflection loss in decibels (dB):

(4.1)Δs21=S21ref−S21mix,(4.2)Δs11=S11ref−S11mix,

where $S_{21}^{ref}$ corresponds to the insertion loss for the sensor with a free surface, and $S_{21}^{mix}$ represents the insertion loss for the sensor when 200 µl of the liquid under analysis is deposited on its surface. Similarly, $S_{11}^{ref}$ denotes the return loss for the sensor with a free surface, whereas $S_{11}^{mix}$ refers to the return loss for the sensor with the liquid under test. Table 7 presents the values of these parameters for the mixtures analysed in this study.

**Table 7.** Relative parameters for samples analysed.

	gasoline	gasoline with 5% anhydrous ethanol	gasoline with 30% anhydrous ethanol	gasoline with 40% anhydrous ethanol	anhydrous ethanol
relative variation in insertion losses (dB)	−5.9	−6.04	−8.62	−9.6	−10.63
relative variation in the magnitude of *S*₁₁ (dB)	−0.13	−0.16	−0.68	−0.81	−1.1

The results presented in table 7 further substantiate the multiphysical signature detected by the sensor. A relative increase in insertion loss is observed for liquids with higher density and viscosity, with a variation of −4.73 dB from anhydrous ethanol to gasoline. This measurable change demonstrates the sensor's ability to resolve differences among the investigated mixtures. Similarly, the relative reflection losses exhibit a systematic variation with increasing liquid permittivity, reaching −0.97 dB from anhydrous ethanol to gasoline. The gradual and correlated changes in these electrical responses with the physicochemical properties of the mixtures provide a robust multiparametric signature that can be exploited to discriminate among different compositions and identify potential deviations from the expected mixture characteristics.

## 5. Conclusion

In this study, a Love-wave sensor with a central frequency of approximately 117.5 MHz was employed to distinguish liquid samples of commercial fuels mixed in different volumetric proportions. This distinction arises from the changes in the electrical and mechanical properties of the liquids, which in turn modify the electroacoustic response of the sensor. It was observed that the magnitude of the insertion loss and the magnitude of the return loss were the most affected parameters. This study demonstrates the great potential of Love-wave sensors for the preliminary analysis of contaminated or irregularly mixed liquids. The proposed method stands out for its simplicity of operation, fast response and portability, enabling on-site fuel quality assessment and providing an almost instantaneous indication of non-conformity before sending samples for laboratory testing, which is often costly, time-consuming and requires specialized personnel.

In this work, it was not possible to assess the measurement uncertainty or the achievable accuracy of the proposed method. Nevertheless, these aspects will be addressed in future studies, contributing to the further development and optimization of Love-wave sensor systems.

Thus, Love-wave sensors should be regarded not as direct replacements for established laboratory methods, but as complementary technologies. Although gas chromatography, molecular spectroscopy and standardized American Society for Testing and Materials (ASTM) methods remain essential for regulatory certification and detailed chemical characterization, Love-wave sensors offer rapid response, miniaturization, low power consumption, continuous reagent-free operation and real time monitoring capabilities. These attributes position technology as a promising solution for rapid screening, field-based quality assessment and preliminary fuel adulteration detection, with the potential to reduce operational costs and enable more frequent quality-control inspections.

## Data Availability

All data used in this study were obtained from experiments conducted by the authors themselves.

## References

[1] Wiedemann LSM , D'Avila LA, Azevedo DA. 2005 Adulteration detection of Brazilian gasoline samples by statistical analysis. Fuel 84, 467–473. (doi:10.1016/j.fuel.2004.09.013)

[2] Pedroso MP , de Godoy LAF, Ferreira EC, Poppi RJ, Augusto F. 2008 Identification of gasoline adulteration using comprehensive two-dimensional gas chromatography combined to multivariate data processing. J. Chromatogr. A 1201, 176–182. (doi:10.1016/j.chroma.2008.05.092) 18571187

[3] Dias GP *et al.* 2020 Determination of methanol in gasoline and ethanol fuels by high-performance liquid chromatography. J. Braz. Chem. Soc. 31, 1055–1063. (doi:10.21577/0103-5053.20190272)

[4] Micyus NJ , McCurry JD, Seeley JV. 2005 Analysis of aromatic compounds in gasoline with flow-switching comprehensive two-dimensional gas chromatography. J. Chromatogr. A 1086, 115–121. (doi:10.1016/j.chroma.2005.06.015) 16130662

[5] Ma W , Halvorsen RC, Cain CN, Synovec RE. 2024 Fuel property modelling by high-speed gas chromatography coupled with partial least squares data analysis. J. Chromatogr. A 1732, 465 220. (doi:10.1016/j.chroma.2024.465220) 39106664

[6] Poole CF . 2015 Ionization-based detectors for gas chromatography. J. Chromatogr. A 1421, 137–153. (doi:10.1016/j.chroma.2015.02.061) 25757823

[7] Mabood F *et al.* 2017 Detection and estimation of Super Premium 95 gasoline adulteration with Premium 91 gasoline using NIR spectroscopy combined with multivariate methods. Fuel 197, 388–396. (doi:10.1016/j.fuel.2017.02.041)

[8] Mishra P , Marini F, Biancolillo A, Roger JM. 2021 Improved prediction of fuel properties with near-infrared spectroscopy using complementary sequential fusion of scatter correction techniques. Talanta 223, 121 693. (doi:10.1016/j.talanta.2020.121693) 33303145

[9] Shrestha B , Posom J, Pornchaloempong P, Sirisomboon P, Shrestha BP, Ariffin H. 2024 Near-infrared spectroscopy modelling of combustion characteristics in chip and ground biomass from fast-growing trees and agricultural residue. Energies 17, 1338. (doi:10.3390/en17061338)

[10] Yankova Y , Cole MD, Cirstea S, Warren J. 2024 Individualisation of petrol sources by high-field nuclear magnetic resonance spectroscopy. Forensic Sci. Int. 361, 112103. (doi:10.1016/j.forsciint.2024.112103) 38901059

[11] Pontes MJC , Santos SRB, Araújo MCU, Almeida LF, Lima RAC, Gaião EN, Souto UTCP. 2006 Classification of distilled alcoholic beverages and verification of adulteration by near infrared spectrometry. Food Res. Int. 39, 182–189. (doi:10.1016/j.foodres.2005.07.005)

[12] Romanel SA , Cunha DA, Castro EV, Barbosa LL. 2018 Time domain nuclear magnetic resonance (TD-NMR): a new methodology to quantify adulteration of gasoline. Microchem. J. 140, 31–37. (doi:10.1016/j.microc.2018.03.041)

[13] Cunha DA , Montes LF, Castro EV, Barbosa LL. 2016 NMR in the time domain: a new methodology to detect adulteration of diesel oil with kerosene. Fuel 166, 79–85. (doi:10.1016/j.fuel.2015.10.078)

[14] Pryde S , Gilbert MT. 1979 Applications of high performance liquid chromatography. Berlin, Germany: Springer Science & Business Media.

[15] Vempatapu BP , Kumar J, Upreti B, Kanaujia PK. 2024 Application of high-performance liquid chromatography in petroleum analysis: challenges and opportunities. TrAC Trends Anal. Chem. 177, 117 810. (doi:10.1016/j.trac.2024.117810)

[16] Rahad R , Rakib AKM, Mahadi MK, Faruque MO. 2023 Fuel classification and adulteration detection using a highly sensitive plasmonic sensor. Sens. Bio-Sens. Res. 40, 100 560. (doi:10.1016/j.sbsr.2023.100560)

[17] Korn M , Santos D, Welz B, Vale M, Teixeira A, Lima D, Ferreira S. 2007 Atomic spectrometric methods for the determination of metals and metalloids in automotive fuels: a review. Talanta 73, 1–11. (doi:10.1016/j.talanta.2007.03.036) 19071843

[18] Nespeca MG , Munhoz J, Flumignan DL, de Oliveira JE. 2018 Rapid and sensitive method for detecting adulterants in gasoline using ultra-fast gas chromatography and partial least square discriminant analysis. Fuel 215, 204–211. (doi:10.1016/j.fuel.2017.11.032)

[19] Silva AC , Paz JEM, Pontes LFBL, Lemos SG, Pontes MJC. 2013 An electroanalytical method to detect adulteration of ethanol fuel using multivariate analysis. Electrochim. Acta 111, 160–164. (doi:10.1016/j.electacta.2013.07.208)

[20] Skrobot VL , Castro EVR, Pereira RCC, Pasa VMD, Fortes ICP. 2005 Identification of gasoline adulteration applying multivariate data analysis techniques HCA and KNN in chromatographic data. Energy Fuels 19, 2350–2356. (doi:10.1021/ef050031l)

[21] ASTM International . 2024 ASTM D4814-24: standard specification for automotive spark-ignition engine fuel. West Conshohocken, PA: ASTM International. (doi:10.1520/D4814-24)

[22] ASTM International . 2024 ASTM D975-24: standard specification for diesel fuel oils. West Conshohocken, PA: ASTM International. (doi:10.1520/D0975-24)

[23] Irshad M , Mujahid A, Afzal A, Bajwa SZ, Hussain T, Zaman WU, Latif U, Athar MM. 2018 A miniaturized electronic sensor for instant monitoring of ethanol in gasohol fuel blends. RSC Adv. 8, 22 952–22 962. (doi:10.1039/C8RA02170H) PMC9081555 35540134

[24] Zhong Y , Huang W, Zhang C, Zhang R, Hu Y, Xiao X, Li G. 2022 Study of cyclic cataluminescence virtual sensor array for gasoline quality monitoring. Sens. Actuators B 364, 131901. (doi:10.1016/j.snb.2022.131901)

[25] Rodríguez-Rodríguez AJ , Domínguez-Cruz RF, May-Arrioja DA. 2017 Liquid fuel identification using a fiber optic sensor based in multimode interference effects (MMI). Res. Comput. Sci. 131, 79–86. (doi:10.13053/rcs-131-1-11)

[26] Mandal D , Banerjee S, Joanni E. 2022 Surface acoustic wave (SAW) sensors: physics, materials, and applications. Sensors 22, 820. (doi:10.3390/s22030820) PMC8839725 35161565

[27] Li X , Wang X, Fu C, Fu Y. 2023 Advances in sensing mechanisms and micro/nanostructured sensing layers for surface acoustic wave-based gas sensors. J. Mater. Chem. A 11, 23 756–23 784. (doi:10.1039/D2TA10014B)

[28] Lin Q , Zhao C, Li M, Xu H. 2024 Recent progress in surface acoustic wave sensors based on low-dimensional materials. Chemosensors 12, 255. (doi:10.3390/chemosensors12120255)

[29] Ramaraj SG , Alrebh A, Elamaran D, Zhou H, Huang K, Almansoori M, Yamahara H, Tabata H. 2025 Surface acoustic wave gas sensors: recent developments and their role in sensing technology. Mater. Sci. Eng. B 317, 118157. (doi:10.1016/j.mseb.2025.118157)

[30] Rapp BE , Voigt A, Dirschka M, Rapp M, Länge K. 2024 Surface acoustic wave resonator chip setup for the elimination of interfering conductivity responses. Micromachines 15, 501. (doi:10.3390/mi15040501) PMC11052277 38675312

[31] Balashov SM , Rocha JM, Hurtado MRF, Prestes JAL, de Campos AFM, Moshkalev SA. 2021 Improved stability and performance of surface acoustic wave nanosensors using a digital temperature compensation. Front. Sens. 2, 617 484. (doi:10.3389/fsens.2021.617484)

[32] Tamarin O , Comeau S, Déjous C, Moynet D, Rebière D, Bezian J, Pistré J. 2003 Real-time device for biosensing: design of a bacteriophage model using Love acoustic waves. Biosens. Bioelectron. 18, 755–763. (doi:10.1016/S0956-5663(03)00022-8) 12706589

[33] Zimmermann C , Rebière D, Déjous C, Pistré J, Chastaing E, Planade R. 2001 A love-wave gas sensor coated with functionalized polysiloxane for sensing organophosphorus compounds. Sens. Actuators B 76, 86–94. (doi:10.1016/S0925-4005(01)00578-0)

[34] Liu Bo , Chen X, Cai H, Mohammad Ali M, Tian X, Tao L, Yang Yi, Ren T. 2016 Surface acoustic wave devices for sensor applications. J. Semicond. 37, 021 001. (doi:10.1088/1674-4926/37/2/021001)

[35] Gongi W , Mougin K, Rube M, Dejous C, Ouada HB, Tamarin O. 2025 Advanced Love wave sensor based on algal polymers for the detection of mercury in French Guiana waters. Sci. Rep. 15, 21 784. (doi:10.1038/s41598-025-99494-8) PMC12216591 40596407

[36] Rube M , Tamarin O, Sebeloue M, Sadli I, Hallil H, Linguet L, Rebiere D, Dejous C. 2021 Unconventional protocol for SAW sensor: multi-physic response enrichment in liquid medium. IEEE Sensors J. 22, 11 345–11 354. (doi:10.1109/jsen.2021.3094299)

[37] Du J , Harding GL, Collings AF, Dencher PR. 1997 An experimental study of love-wave acoustic sensors operating in liquids. Sens. Actuators A 60, 54–61. (doi:10.1016/s0924-4247(96)01424-0)

[38] Bernardes DJP , Santana EEC, Freire RCS, Júnior P, Sebeloue M. 2025 Electrical model of a Love wave acoustic sensor with resonance frequency of 117 MHz. In 2025 9th Int. Symp. on Instrumentation Systems, Circuits and Transducers (INSCIT), pp. 1–6. Piscataway, NJ: IEEE. (doi:10.1109/INSCIT66472.2025.11180992)

[39] Tamarin O , Déjous C, Rebière D, Pistré J, Comeau S, Moynet D, Bezian J. 2003 Study of acoustic Love wave devices for real-time bacteriophage detection. Sens. Actuators B 91, 275–284. (doi:10.1016/s0925-4005(03)00106-0) 12706589

